# In Vivo Reflectance Confocal Microscopy-Diagnostic Criteria for Actinic Cheilitis and Squamous Cell Carcinoma of the Lip

**DOI:** 10.3390/jcm9061987

**Published:** 2020-06-25

**Authors:** Mihai Lupu, Ana Caruntu, Daniel Boda, Constantin Caruntu

**Affiliations:** 1 Dermatology Research Laboratory, “Carol Davila” University of Medicine and Pharmacy, 050474 Bucharest, Romania; lupu.g.mihai@gmail.com (M.L.); daniel.boda@yahoo.com (D.B.); 2 Department of Oral and Maxillofacial Surgery, “Carol Davila” Central Military Emergency Hospital, 010825 Bucharest, Romania; 3 Faculty of Medicine, “Titu Maiorescu” University, 031593 Bucharest, Romania; 4 Department of Dermatology, “Prof. N.C. Paulescu” National Institute of Diabetes, Nutrition and Metabolic Diseases, 011233 Bucharest, Romania; costin.caruntu@gmail.com; 5 Department of Physiology, “Carol Davila” University of Medicine and Pharmacy, 050474 Bucharest, Romania

**Keywords:** actinic cheilitis, squamous cell carcinoma, in vivo, reflectance confocal microscopy, lip neoplasms

## Abstract

Actinic cheilitis (AC) is one of the most frequent pathologies to affect the lips. Studies show that the most commonplace oral malignancy, squamous cell carcinoma (SCC), often emerges from AC lesions. Invasive diagnostic techniques performed on the lips carry a high risk of complications, but reflectance confocal microscopy (RCM), a non-invasive skin imaging technique, may change the current diagnostic pathway. This retrospective study was aimed at consolidating the RCM diagnostic criteria for AC and lip SCC. The study was conducted in two tertiary care centers in Bucharest, Romania. We included adults with histopathologically confirmed AC and SCC who also underwent RCM examination. Of the twelve lesions included in the study, four were AC and eight were SCC. An atypical honeycomb pattern and the presence of target cells in the epidermis were RCM features associated with AC. SCC was typified by the presence of complete disruption of the epidermal architecture and dermal inflammatory infiltrates. The mean blood vessel diameter in SCC was 18.55 µm larger than that in AC (*p* = 0.006) and there was no significant difference (*p* = 0.64) in blood vessel density, as measured by RCM, between SCC and AC. These data confirm that RCM can be useful for the *in vivo* distinction between AC and lip SCC.

## 1. Introduction

Lips constitute a special location for the development of numerous skin lesions due to their frequent exposure to exogenous factors, such as ultraviolet light, chemical, and biological agents.

Actinic cheilitis (AC) is one of the most often occurring pathologies that affects the lips [1]. Occupational activities not considered, studies report an AC prevalence between 0.2% and 0.45% [2,3].

Upon clinical examination, AC has a broad spectrum of presentation, comprised of pale, dry, scaly lips, chronic ulcerations and erosions [4], blurring of the vermillion-skin border [2,5,6,7,8,9,10], white [11,12] and red [2,5,9,10] areas, and vermillion atrophy [5,6,7,8]. Palpation reveals a fine sandpaper-like feeling [4], often accompanied by a stinging or burning and an inelastic or tight sensation of the lip [4,8].

AC differential diagnosis includes inflammatory disorders such as eczema, benign leukoplakia, lichen planus, granulomatous cheilitis, and xerosis with chronic irritation [13,14]. 

Some studies show that squamous cell carcinoma (SCC) represents approximately 90% of all oral malignancy cases [15] while others estimate that 95% of SCCs of the lips emerge from ACs [12,16].

Changes suggestive of AC progression to SCC of the lip include thickening and induration of keratotic AC patches, the appearance of nodules with rapid growth and/or ulceration associating bleeding and pain [4,8,17,18,19,20]. Lip squamous cell carcinoma is also much more prone to metastasis than cutaneous SCC (0.5–3% vs. 3–20%) [21,22,23].

A number of techniques hold promise for the early detection, aggressiveness profile and monitoring of keratinocyte carcinomas. The observed differences between normal, inflammatory and malignant keratinocyte proteomic profiles are likely to unearth novel markers for SCC, in terms of diagnosis and monitoring, and could maybe even come to the aid of targeted therapies [24,25,26,27].

Although the gold standard diagnostic technique for AC and lip SCC is the histopathological examination of a biopsy specimen, the anatomic characteristics of the lips increase the risk of postoperative bleeding and infection. Additionally, considering the cosmetic importance of this area, noninvasive diagnostic techniques are useful for selecting the biopsy site, thus avoiding repeated biopsies, and in some cases even acting as a surrogate for histopathology.

Imaging techniques, such as dermoscopy and *in vivo* reflectance confocal microscopy (RCM), continue to highlight diagnostic and prognostic criteria for AC and SCC [28,29]. The lips are an ideal site for RCM examination, due to a thinner epidermis when compared to other body sites. Because early detection and swift therapy remain the two most important factors influencing the long-term survival of these patients [30], we designed a retrospective study with the aim of consolidating previous observations regarding the RCM diagnostic criteria for AC and lip SCC.

## 2. Materials and Methods

### 2.1. Subjects

Patients with histopathologically confirmed lesions of actinic cheilitis or lip SCC were included in the study. Patients’ records were retrieved from the electronic database of the Dermatology Research Laboratory, “Carol Davila” University of Medicine and Pharmacy, in Bucharest. The study was conducted in accordance with the Declaration of Helsinki, and the protocol was approved by the Local Ethics Committee (No. 25/27.11.2017). All participants gave written informed consent as part of their investigation and treatment procedures, at the time of their registration.

### 2.2. RCM Imaging and Analysis

Despite it being a retrospective study, the RCM imaging protocol was the same for all lesions, as it is a well-established protocol in this clinic when examining non-melanocytic lesions. 

Confocal imaging was done with a commercially available confocal microscope (Vivascope^®^ 1500, Caliber ID, Rochester, NY, USA) which uses a near-infrared laser diode with a maximum power output of 20 mW. The device and image acquisition protocol have been described elsewhere [31].

Vertical imaging via Vivastack^®^ was performed by capturing a series of images of 0.5 × 0.5 mm with 3 µm increments, in depth. Horizontal mosaics (via Vivablock^®^) of 4 × 4 mm were captured at different depths of the lesions. Mapping started at the stratum corneum and was continued to the papillary dermis.

Due to the problematic separation of epidermal layers on RCM, we adopted the methodology of Peppelman et al. [32], so that the first appearance of nucleated cells, regardless of cell size and shape, was considered to be the stratum granulosum (SG). Since the SG is only a few cell layers thick, three steps in depth below this point was considered as the stratum spinosum (SS). 

Diagnostic RCM criteria for AC and lip SCC were selected based on previously published data (Table 1) [33,34,35,36,37,38,39,40,41,42]. 

RCM images were then evaluated for these features by an RCM user (LM) with 4 years of experience with this technique. The observer systematically evaluated the lesions for the presence or absence of individual RCM criteria, but was not blinded to the histopathological diagnosis. Furthermore, the mean vessel diameter and blood vessel density per individual confocal image (500 × 500 µm) were determined for both AC and lip SCC lesions. Based on previously published data [32], an increased vascular diameter was defined as a diameter greater than 5 µm and an increased blood vessel density as more than 5 vessels per single confocal image (500 × 500 µm). Blood vessel diameter measurements were taken on scale calibrated images using the open source software package ImageJ. A line, perpendicular to the axis of the vessel, was drawn from side to side in the widest visible part of the blood vessel and the result was recorded. This measurement was repeated for every visible vessel in the chosen RCM image. For every case, these two parameters were measured in 3 different single confocal images at approximately the same depth for every lesion, and the highest value for each of these two parameters was recorded for the case.

### 2.3. Histopathology 

All lesions included in this study were surgically excised after RCM investigation and histologically confirmed as either AC or lip SCC by an experienced pathologist on haematoxylin–eosin (H&E) stained paraffin sections. SCC was defined based on the presence of an invasive component.

### 2.4. Statistical Analysis

The analysis was conducted in order to assess how various observed RCM criteria were associated with either AC or SCC. It comprised descriptive statistics and Fisher’s exact test to analyze the differences between subgroups. The differences in blood vessel diameter and vessel density per individual confocal image in AC and SCC lesions were measured using an independent t-test.

All data analyses were conducted using the statistical software package SPSS Inc. (v20, Chicago, IL, USA).

## 3. Results

Twelve subjects (10 males and 2 females) with a mean age of 66.82 ± 9.87 (range 43–80) years were included in the study.

In these patients, a total of 12 biopsy-proven lesions were evaluated with RCM, of which four ACs and eight invasive lip SCCs. The lesions were all located on the vermillion of the lower lip. In the actinic cheilitis subgroup, there were two smokers (one male and one female) and in the SCC subgroup there were three smokers (all male). None of the subjects included in this study were immunosuppressed.

The degree of differentiation for the SCC lesions included in the study, along with gender, age, immune and smoking status, are illustrated in Table 2.

### 3.1. RCM Features for Differentiating between AC and Lip SCC

**Figure 1:**
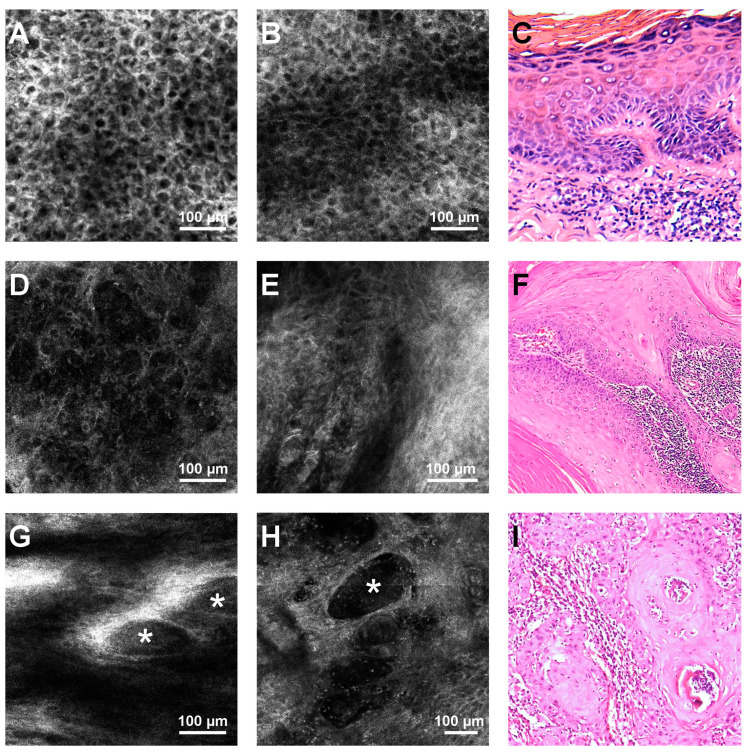
Representative reflectance confocal microscopy (RCM) images of actinic cheilitis (AC) and lip squamous cell carcinoma (SCC), and histological correspondents. (**A**) RCM image of an atypical honeycomb pattern of the stratum granulosum, seen in an AC lesion. (**B**) RCM image at the stratum spinosum showing an atypical honeycomb pattern, which can be seen in either AC or SCC. (**C**) Histopathology image illustrating parakeratosis, atypical keratinocytes in the stratum granulosum and spinosum, spongiosis, and intradermal inflammatory infiltrate in an AC lesion (haematoxylin-eosin, cropped, original magnification 40×). (**D**) RCM image of the complete architectural disarray in the granular layer of a lip SCC. (**E**) RCM image showing disarray in the stratum spinosum, in a SCC lesion. (**F**) Histopathological image displaying infiltrative atypical polygonal squamous cells with distinct cell borders, abundant eosinophilic cytoplasm, and large vesicular nuclei with moderate nuclear pleomorphism in a SCC (haematoxylin-eosin, cropped, original magnification 40×) (**G**,**H**). RCM images showing tumor nests (white asterisks) surrounded by white areas corresponding to fibrosis at the level of the dermis, in a SCC. (**I**) Histopathology image illustrating invasive SCC nests and strands of atypical polygonal squamous cells surrounded by intradermal inflammatory infiltrate in a lip SCC (haematoxylin–eosin, cropped, original magnification 100×).

Hyperkeratosis (4/4), parakeratosis (3/4), an atypical honeycomb pattern (4/4), and the presence of dyskeratotic, targetoid cells within the epidermis (4/4) were RCM features present in virtually all the examined AC lesions (Figure 1). Total epidermal disarray, dendritic and atypical cells in the dermis, and tumor nests in the dermis were found in none of the AC lesions.

Lip SCCs were typified in confocal examination by the presence of the complete disruption of the epidermal architecture (8/8) and the presence of dermal inflammatory infiltrate (6/8). Atypical cells in the dermis and dermal tumor nests could be detected upon RCM examination in only half of the SCC lesions (Figure 1). Dendritic cells, most probably corresponding to Langerhans cells, were seen in three out of eight SCCs. Although in SCCs with a pigmentary component, melanocytes can be seen in RCM, none of the tumors included in our study had a clinically or dermatoscopically visible pigmentary component. Even so, we cannot exclude the possibility that some of the observed dendritic cells could indeed be melanocytes.

Ulceration, hyperkeratosis/scale, and dermal solar elastosis were present in both AC and SCC with similar frequencies.

Table 3 shows the frequencies of the various RCM criteria in the studied AC and lip SCC lesions. Table 4 contains the frequencies of the observed RCM criteria according to SCC degree of differentiation. None of the RCM criteria varied significantly between well and moderately differentiated SCC, as assessed by the Chi-square test.

**Figure 2:**
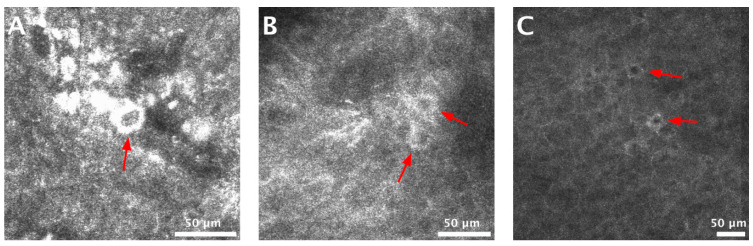
Target cells in the epidermis of actinic cheilitis (AC) lesions. (**A**–**C**) Reflectance confocal microscopy (RCM) images showing target cells (red arrows), corresponding to dyskeratotic keratinocytes, at the level of the epidermis in AC lesions.

The assessment of associations between RCM criteria and the diagnosis of AC or lip SCC revealed that an atypical honeycomb pattern (*p* = 0.002, Fisher’s exact test) and the presence of target keratinocytes in the epidermis (*p* = 0.01, Fisher’s exact test) were strongly associated with AC (Figure 2), while complete epidermal disarray (*p* = 0.002, Fisher’s exact test) was characteristic for lip SCC. The RCM features for AC/SCC discrimination in this study have been summarized in Table 5.

### 3.2. Vascularization in AC and SCC Lesions

**Figure 3:**
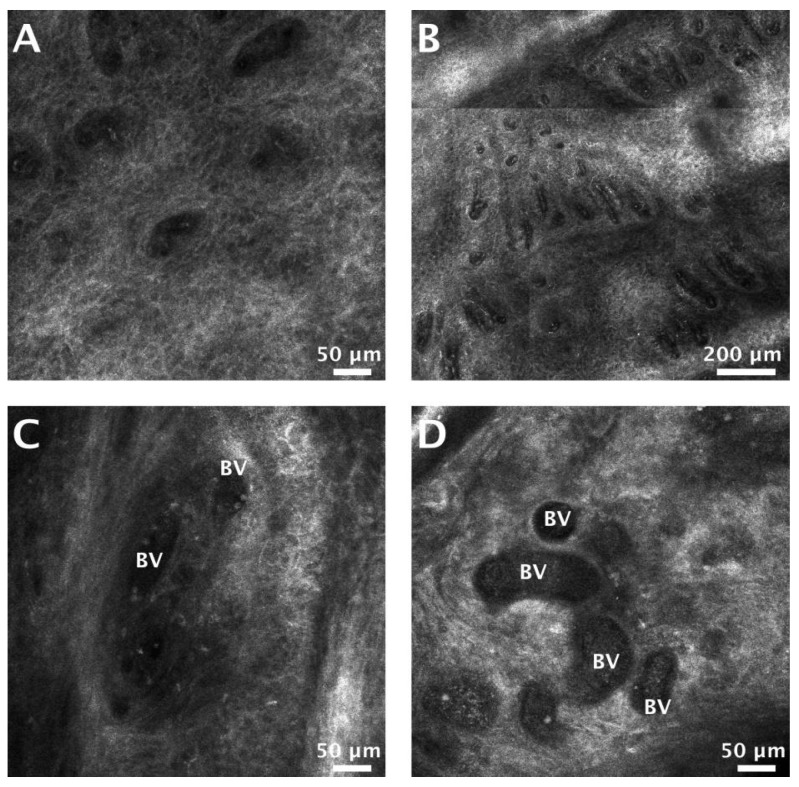
Blood vessel density and blood vessel dilation in actinic cheilitis (AC) and lip squamous cell carcinoma (SCC). (**A**) Reflectance confocal microscopy (RCM) image showing blood vessels (dark areas in the honeycomb) at the level of the dermal-epidermal junction (DEJ), in an AC lesion. (**B**) RCM mosaic (1305 × 1305 µm) displaying a high density of blood vessels at the DEJ in a SCC. (**C**) RCM image of dilated blood vessels (BV) filled with moderately-refractile particles (corresponding to blood cells) in an AC lesion. (**D**) RCM image showing markedly dilated blood vessels (BV) at dermal level in a SCC.

The vessel diameter and number of blood vessels per single RCM image were higher in SCC (Figure 3). The mean blood vessel diameter in SCCs was 18.55 µm larger than that in AC lesions (*p* = 0.006). There was no significant difference (*p* = 0.64) in blood vessel density, as measured by RCM, between SCC and AC lesions in our sample (Table 6).

## 4. Discussions

Aside from the particular functional and cosmetic significance of the lips, several conditions ranging from benign infections to dysplasias, and the potentially fatal SCC, may develop in this area. Hence, the examination of the vermillion and lip mucosa is an important part of the dermatological examination.

While there are numerous publications regarding RCM imaging of various skin conditions ranging from tumors to infections and inflammatory conditions [33,34,35,43,44,45,46,47], there are only a few reports in the literature related to the non-invasive diagnosis of lip lesions. Whilst RCM knowledge and experience in the field of non-melanoma skin cancer is constantly expanding [48,49,50,51], obtaining biopsies is still an invasive procedure with its own limitations, mainly due to cosmetic reasons and the risk of sampling errors.

An obvious limitation of our study is the small sample size of only 12 lesions. It remains difficult to have a large sample size of SCCs in non-invasive diagnostic studies. The hyperkeratotic scale makes the evaluation of SCC difficult with either dermoscopy or RCM, hence the limited advantage of these techniques in clinically evident SCCs. Additionally worth mentioning is the difficulty raised by the need for histopathological confirmation in this type of study. The most important struggle in this field remains in distinguishing between SCC in situ and invasive SCC in the case of clinically similar lesions.

Ulrich et al. [41] defined the RCM criteria for AC as: disruption of stratum corneum, hyperkeratosis, parakeratosis, atypical honeycomb pattern at the SS and SG, dermal solar elastosis, dilated blood vessels, and the presence of inflammatory cells in the upper dermis. In our study, the most common RCM feature for both AC and SCC was the presence of keratinocyte pleomorphism resulting in either an atypical honeycomb pattern in the case of AC or a complete disruption of the epidermal architecture in SCC. Furthermore, testing for the association between RCM criteria and these two entities, we uncovered that target cells are significantly associated with AC. These findings are in accordance with previous studies which examined the RCM appearance of actinic keratoses and cutaneous SCCs [32,38,52,53].

As opposed to previous studies [54], dermal dyskeratotic keratinocytes were observed in only 50% of SCC lesions included in our study, either as isolated, scattered cells or as tumor nests. However, similar to earlier research [54], we found dermal dendritic cells in under half of the lip SCCs (3/8) and these results do not allow for a significant association between this element and lip SCC. On the other hand, we observed the presence of inflammatory cells in the dermis in half of the ACs and almost all (6/8) of the SCCs, which is in line with other studies [54], and reveals traits of the tumor microenvironment [55]. While not statistically significant, Hartmann et al. [56] reported an increased frequency of RCM-observed dermal tumor nests and peritumoral inflammatory infiltrates in moderately differentiated SCCs compared to well differentiated SCCs. In our data, none of the RCM criteria were significantly different between well and moderately differentiated SCC. In our case, we attribute this to the small sample size.

When analyzing AC and lip SCC vasculature, we found a significantly increased mean vascular diameter and a larger blood vessel density for SCC compared to AC. Our results are in accordance with other studies, and can be explained by the high metabolic needs of a tumor, which determines vascular dilation and neovascularization [32,57,58].

To conclude, this study, building upon previous research, confirms several RCM criteria which can be used to distinguish between AC and lip SCC in vivo. This warrants further prospective, large sample-size studies, which will form the basis for the development of protocols for the correct, efficient and expeditious diagnosis of AC and SCC of the lips.

## Figures and Tables

**Table 1:** Reflectance confocal microscopy (RCM) criteria for the diagnosis of actinic cheilitis (AC) and lip squamous cell carcinoma (SCC).

**Epidermis**	
Ulceration	Dark areas, with irregular and well-defined borders, filled with amorphous material and cellular debris.
Hyperkeratosis/scale	Increased thickness of the stratum corneum seen as areas of amorphous, variably refractive material, and reduced resolution of deeper structures.
Parakeratosis	Presence of individual polygonal, sharply delineated, nucleated cells in the stratum corneum.
Atypical honeycomb pattern SG/SS *	Cells with irregular shape and size showing bright cell borders, arranged in a distorted fashion, deviating from the normal honeycomb pattern.
Architectural disarrangement SG/SS*	Disarray of the normal architecture of the superficial skin layers with unevenly dispersed hyper-refractive granular particles and cells, in which the honeycomb or cobblestone patterns are no longer visible.
Targetoid cells SS/SG*	A large cell resembling a target, either with a bright center and dark peripheral halo or a dark center and a bright rim surrounded by a dark peripheral halo. The first one corresponds histologically to large dyskeratotic keratinocytes separated from adjacent cells by a retraction halo, and the second type to dyskeratotic keratinocytes containing a pyknotic nucleus.
Dendritic cells	Large cells with obvious dendrites connected to them.
**Dermal-epidermal junction**	
Increased vessel diameter	Blood vessel diameter larger than 5 µm.
Increased vessel density	More than 5 blood vessels per 0.5 × 0.5 mm RCM image.
**Dermis**	
Solar elastosis	Lace-like material adjacent to hyper-refractive, thickened collagen bundles.
Inflammatory cells	Hyper-refractive, small structures, of 8–10 µm in diameter.
Dendritic cells	Large cells with obvious dendrites connected to them.
Atypical keratinocytes (speckled/nucleated)	Round to polygonal cells with a dark nucleus and speckled appearance.
Nest-like structures	Defined, irregular, discohesive, aggregates of cells larger than inflammatory cells.
Keratin pearls	Whorl-shaped, hyper-refractive, speckled structures.

* SG/SS, stratum granulosum/stratum spinosum.

**Table 2:** Degree of differentiation, immune and smoking status in the SCC subgroup.

Sex	Age	Smoking	Immune Status	SCC Degree of Differentiation
male	43	yes	immunocompetent	moderately differentiated
female	59	no	immunocompetent	well differentiated
male	80	no	immunocompetent	well differentiated
male	69	no	immunocompetent	moderately differentiated
male	71	yes	immunocompetent	well differentiated
male	66	no	immunocompetent	moderately differentiated
male	68	yes	immunocompetent	moderately differentiated
male	65	no	immunocompetent	moderately differentiated

SCC, squamous cell carcinoma.

**Table 3:** Frequencies of RCM criteria for AC and lip SCC.

RCM Criteria, N (%)	Histopathological Diagnosis	
	AC (N = 4)	Lip SCC (N = 8)
Ulceration	3 (75%)	7 (87.5%)
Hyperkeratosis/scale	4 (100%)	7 (87.5%)
Parakeratosis	3 (75%)	3 (37.5%)
Atypical honeycomb pattern	4 (100%)	0 (0%)
Epidermal disarray	0 (0%)	8 (100%)
Target cells in the epidermis	4 (100%)	1 (12.5%)
Dendritic cells in the epidermis	1 (25%)	0 (0%)
Solar elastosis	2 (50%)	5 (62.5%)
Dermal inflammatory cells	2 (50%)	6 (75%)
Dendritic cells in the dermis	0 (0%)	3 (37.5%)
Atypical cells in the dermis	0 (0%)	4 (50%)
Tumor nests in the dermis	0 (0%)	4 (50%)

RCM, reflectance confocal microscopy; AC, actinic cheilitis; SCC, squamous cell carcinoma.

**Table 4:** Frequencies of RCM criteria according to SCC degree of differentiation.

RCM Criteria, N (%)	Squamous Cell Carcinoma		*p*
	Well Differentiated (N = 3)	Moderately Differentiated (N = 5)	
Ulceration	3 (100%)	4 (80%)	0.408
Hyperkeratosis/scale	3 (100%)	4 (80%)	0.408
Parakeratosis	0 (0%)	3 (60%)	0.09
Atypical honeycomb pattern	0 (0%)	0 (0%)	-
Epidermal disarray	3 (100%)	5 (100%)	-
Target cells in the epidermis	1 (33.3%)	0 (0%)	0.168
Dendritic cells in the epidermis	0 (0%)	0 (0%)	-
Solar elastosis	2 (66.7%)	3 (60%)	0.85
Dermal inflammatory cells	3 (100%)	3 (60%)	0.206
Dendritic cells in the dermis	0 (0%)	3 (60%)	0.09
Atypical cells in the dermis	2 (66.7%)	2 (40%)	0.465
Tumor nests in the dermis	2 (66.7%)	2 (40%)	0.465

RCM, reflectance confocal microscopy; SCC, squamous cell carcinoma.

**Table 5:** Specific RCM features associated with AC and lip SCC.

RCM Criteria	*p*
**Actinic cheilitis**	
Atypical honeycomb pattern	0.002
Target cells in the epidermis	0.01
**Lip squamous cell carcinoma**	
Complete epidermal disarray	0.002

AC, actinic cheilitis; SCC, squamous cell carcinoma; RCM, reflectance confocal microscopy.

**Table 6:** Blood vessels characteristics of patients with AC and lip SCC.

	Histopathological Diagnosis		*p*
	AC	Lip SCC	
	Mean ± SD	Mean ± SD	
Mean blood vessel diameter (µm)	19.26 ± 5.67	37.81 ± 12.77	0.006
Mean number of blood vessels	8.25 ± 1.89	8.88 ± 2.53	0.64

AC, actinic cheilits; SCC, squamous cell carcinoma; SD, standard deviation.

## References

[1] De Lucena E.E.S., Costa D.C.B., da Silveira E.J.D., Lima K.C. (2012). Prevalence and factors associated to actinic cheilitis in beach workers. Oral Dis..

[2] Kaugars G.E., Pillion T., Svirsky J.A., Page D.G., Burns J.C., Abbey L.M. (1999). Actinic cheilitis: A review of 152 cases. Oral Surg. Oral Med. Oral Pathol. Oral Radiol Endodontology.

[3] Corso F., Wild C., Gouveia L., Ribas M. (2006). Actinic cheilitis: Prevalence in dental clinics from pucpr, curitiba, brazil. Rev. Clin. Pesq Odontol.

[4] Markopoulos A., Albanidou-Farmaki E., Kayavis I. (2004). Actinic cheilitis: Clinical and pathologic characteristics in 65 cases. Oral Dis..

[5] Cavalcante A.S.R., Anbinder A.L., Carvalho Y.R. (2008). Actinic cheilitis: Clinical and histological features. J. Oral Maxillofac. Surg..

[6] Savage N.W., McKay C., Faulkner C. (2010). Actinic cheilitis in dental practice. Aust. Dent. J..

[7] Vieira R.A.M.A.R., Minicucci E.M., Marques M.E.A., Marques S.A. (2012). Actinic cheilitis and squamous cell carcinoma of the lip: Clinical, histopathological and immunogenetic aspects. An. Bras. Dermatol..

[8] Nico M.M.S., Rivitti E.A., Lourenço S.V. (2007). Actinic cheilitis: Histologic study of the entire vermilion and comparison with previous biopsy. J. Cutan. Pathol..

[9] Miranda A.M., Soares L.G., Ferrari T.M., Silva D.G., Falabella M.E., Tinoco E. (2012). Prevalence of actinic cheilitis in a population of agricultural sugarcane workers. Acta Odontol. Latinoam..

[10] Miranda A.M., Ferrari T., Leite T., Domingos T., Cunha K., Dias E. (2015). Value of videoroscopy in the detection of alterations of actinic cheilitis and the selection of biopsy areas. Med. Oral Patol. Oral Cir. Bucal..

[11] De Sarmento D.J.S., da Miguel M.C.C., Queiroz L.M., Godoy G.P., da Silveira E.J. (2014). Actinic cheilitis: Clinicopathologic profile and association with degree of dysplasia. Int. J. Dermatol..

[12] Lopes M.L., Junior F.L.S., Lima K.C., Oliveira P.T., Silveira E.J. (2015). Clinicopathological profile and management of 161 cases of actinic cheilitis. An. Bras. Dermatol..

[13] Picascia D.D., Robinson J.K. (1987). Actinie cheilitis: A review of the etiology, differential diagnosis, and treatment. J. Am. Acad. Dermatol..

[14] Ulrich M., Gonzalez S., Lange-Asschenfeldt B., Roewert-Huber J., Sterry W., Stockfleth E., Astner S. (2011). Non-invasive diagnosis and monitoring of actinic cheilitis with reflectance confocal microscopy. J. Eur. Acad. Dermatol. Venereol..

[15] Cooper J.S., Porter K., Mallin K., Hoffman H.T., Weber R.S., Ang K.K., Gay E.G., Langer C.J. (2009). National cancer database report on cancer of the head and neck: 10-year update. Head Neck.

[16] Miranda A.M.O., Ferrari T.M., Calandro T.L.L. (2011). Queilite actínica: Aspectos clínicos e prevalência encontrados em uma população rural do interior do brasil. Saúde E Pesquisa.

[17] Cockerell C.J. (2003). Pathology and pathobiology of the actinic (solar) keratosis. Br. J. Dermatol..

[18] Holmes C., Foley P., Freeman M., Chong A.H. (2007). Solar keratosis: Epidemiology, pathogenesis, presentation and treatment. Australas. J. Dermatol..

[19] Wood N.H., Khammissa R., Meyerov R., Lemmer J., Feller L. (2011). Actinic cheilitis: A case report and a review of the literature. Eur. J. Dent..

[20] Kwon N.H., Kim S.Y., Kim G.M. (2011). A case of metastatic squamous cell carcinoma arising from actinic cheilitis. Ann. Dermatol.

[21] de Abreu M.A.M.M., da Silva O.M.P., Pimentel D.R.N., Hirata C.H.W., Weckx L.L.M., de Alchorne M.M.A., Michalany N.S. (2006). Actinic cheilitis adjacent to squamous carcinoma of the lips as an indicator of prognosis. Braz. J. Otorhinolaryngol..

[22] Glogau R.G. (2000). The risk of progression to invasive disease. J. Am. Acad. Dermatol..

[23] Moy R.L. (2000). Clinical presentation of actinic keratoses and squamous cell carcinoma. J. Am. Acad. Dermatol..

[24] Ion A., Popa I.M., Papagheorghe L.M.L., Lisievici C., Lupu M., Voiculescu V., Caruntu C., Boda D. (2016). Proteomic approaches to biomarker discovery in cutaneous t-cell lymphoma. Dis. Markers.

[25] Lupu M., Caruntu C., Ghita M.A., Voiculescu V., Voiculescu S., Rosca A.E., Caruntu A., Moraru L., Popa I.M., Calenic B. (2016). Gene expression and proteome analysis as sources of biomarkers in basal cell carcinoma. Dis. Markers.

[26] Voiculescu V., Calenic B., Ghita M., Lupu M., Caruntu A., Moraru L., Voiculescu S., Ion A., Greabu M., Ishkitiev N. (2016). From normal skin to squamous cell carcinoma: A quest for novel biomarkers. Dis. Markers.

[27] Solomon I., Voiculescu V.M., Caruntu C., Lupu M., Popa A., Ilie M.A., Albulescu R., Caruntu A., Tanase C., Constantin C. (2018). Neuroendocrine factors and head and neck squamous cell carcinoma: An affair to remember. Dis. Markers.

[28] Lupu M., Caruntu A., Caruntu C., Boda D., Moraru L., Voiculescu V., Bastian A. (2018). Non-invasive imaging of actinic cheilitis and squamous cell carcinoma of the lip. Mol. Clin. Oncol..

[29] Lupu M., Căruntu A., Moraru L., Voiculescu V.M., Boda D., Tănase C., Căruntu C. (2018). Non-invasive imaging techniques for early diagnosis of radiation-induced squamous cell carcinoma of the lip. Rom. J. Morphol. Embryol..

[30] Ridgway J.M., Armstrong W.B., Guo S., Mahmood U., Su J., Jackson R.P., Shibuya T., Crumley R.L., Gu M., Chen Z. (2006). In vivo optical coherence tomography of the human oral cavity and oropharynx. Arch. Otolaryngol. Head Neck Surg..

[31] Lupu M., Popa I.M., Voiculescu V.M., Boda D., Caruntu C., Zurac S., Giurcaneanu C. (2019). A retrospective study of the diagnostic accuracy of in vivo reflectance confocal microscopy for basal cell carcinoma diagnosis and subtyping. J. Clin. Med..

[32] Peppelman M., Nguyen K.P., Hoogedoorn L., van Erp P.E.J., Gerritsen M.J.P. (2014). Reflectance confocal microscopy: Non-invasive distinction between actinic keratosis and squamous cell carcinoma. J. Eur. Acad. Dermatol. Venereol..

[33] Guitera P., Menzies S.W., Longo C., Cesinaro A.M., Scolyer R.A., Pellacani G. (2012). In vivo confocal microscopy for diagnosis of melanoma and basal cell carcinoma using a two-step method: Analysis of 710 consecutive clinically equivocal cases. J. Invest. Dermatol..

[34] Langley R.G.B., Walsh N., Sutherland A.E., Propperova I., Delaney L., Morris S.F., Gallant C. (2007). The diagnostic accuracy of in vivo confocal scanning laser microscopy compared to dermoscopy of benign and malignant melanocytic lesions: A prospective study. Dermatology.

[35] Wolberink E.A.W., van Erp P.E.J., Teussink M.M., van de Kerkhof P.C.M., Gerritsen M.J.P. (2010). Cellular features of psoriatic skin: Imaging and quantification using in vivo reflectance confocal microscopy. Cytom. Part B Clin. Cytom..

[36] Horn M., Gerger A., Ahlgrimm-Siess V., Weger W., Koller S., Kerl H., Samonigg H., Smolle J., Hofmann-Wellenhof R. (2008). Discrimination of actinic keratoses from normal skin with reflectance mode confocal microscopy. Dermatol. Surg..

[37] Ulrich M., Forschner T., Röwert-Huber J., González S., Stockfleth E., Sterry W., Astner S. (2007). Differentiation between actinic keratoses and disseminated superficial actinic porokeratoses with reflectance confocal microscopy. Br. J. Dermatol..

[38] Aghassi D., Anderson R.R., Gonzlez S. (2000). Confocal laser microscopic imaging of actinic keratoses in vivo: A preliminary report. J. Am. Acad. Dermatol..

[39] Peppelman M., Wolberink E.A.W., Koopman R.J.J., van Erp P.E.J., Gerritsen M.-J.P. (2013). In vivo reflectance confocal microscopy: A useful tool to select the location of a punch biopsy in a large, clinically indistinctive lesion. Case Rep. Dermatol..

[40] Richtig E., Ahlgrimm-Siess V., Koller S., Gerger A., Horn M., Smolle J., Hofmann-Wellenhof R. (2010). Follow-up of actinic keratoses after shave biopsy byin-vivoreflectance confocal microscopy-a pilot study. J. Eur. Acad. Dermatol. Venereol..

[41] Ulrich M., Lange-Asschenfeldt S., González S. (2012). In vivo reflectance confocal microscopy for early diagnosis of nonmelanoma skin cancer. Actas Dermosifiliogr..

[42] Rajadhyaksha M., González S., Zavislan J.M., Rox Anderson R., Webb R.H. (1999). In vivo confocal scanning laser microscopy of human skin ii: Advances in instrumentation and comparison with histology. J. Invest. Dermatol..

[43] Langley R.G.B., Burton E., Walsh N., Propperova I., Murray S.J. (2006). In vivo confocal scanning laser microscopy of benign lentigines: Comparison to conventional histology and in vivo characteristics of lentigo maligna. J. Am. Acad. Dermatol..

[44] González S., González E., White W.M., Rajadhyaksha M., Anderson R.R. (1999). Allergic contact dermatitis: Correlation of in vivo confocal imaging to routine histology. J. Am. Acad. Dermatol..

[45] Ilie M.A., Caruntu C., Lixandru D., Tampa M., Georgescu S.R., Constantin M.M., Constantin C., Neagu M., Zurac S.A., Boda D. (2019). In vivo confocal laser scanning microscopy imaging of skin inflammation: Clinical applications and research directions. Exp. Ther. Med..

[46] Ilie M.A., Caruntu C., Lupu M., Lixandru D., Georgescu S.-R., Bastian A., Constantin C., Neagu M., Zurac S.A., Boda D. (2019). Current and future applications of confocal laser scanning microscopy imaging in skin oncology. Oncol. Lett..

[47] Ianoși S.L., Forsea A.M., Lupu M., Ilie M.A., Zurac S., Boda D., Ianosi G., Neagoe D., Tutunaru C., Popa C.M. (2019). Role of modern imaging techniques for the in vivo diagnosis of lichen planus. Exp. Ther. Med..

[48] Lupu M., Caruntu C., Popa M.I., Voiculescu V.M., Zurac S., Boda D. (2019). Vascular patterns in basal cell carcinoma: Dermoscopic, confocal and histopathological perspectives (review). Oncol. Lett..

[49] Lupu M., Popa I.M., Voiculescu V.M., Caruntu A., Caruntu C. (2019). A systematic review and meta-analysis of the accuracy of in vivo reflectance confocal microscopy for the diagnosis of primary basal cell carcinoma. J. Clin. Med..

[50] Caruntu C., Boda D., Gutu D.E., Caruntu A. (2014). In vivo reflectance confocal microscopy of basal cell carcinoma with cystic degeneration. Rom. J. Morphol. Embryol..

[51] Ghita M.A., Caruntu C., Rosca A.E., Kaleshi H., Caruntu A., Moraru L., Docea A.O., Zurac S., Boda D., Neagu M. (2016). Reflectance confocal microscopy and dermoscopy for in vivo, non-invasive skin imaging of superficial basal cell carcinoma. Oncol. Lett..

[52] Rishpon A., Kim N., Scope A., Porges L., Oliviero M.C., Braun R.P., Marghoob A.A., Fox C.A., Rabinovitz H.S. (2009). Reflectance confocal microscopy criteria for squamous cell carcinomas and actinic keratoses. Arch. Dermatol..

[53] Braga J.C.T., Scope A., Klaz I., Mecca P., González S., Rabinovitz H., Marghoob A.A. (2009). The significance of reflectance confocal microscopy in the assessment of solitary pink skin lesions. J. Am. Acad. Dermatol..

[54] Bağcı I.S., Gürel M.S., Aksu A.E.K., Erdemir A.T., Yüksel E.İ., Başaran Y.K. (2017). Reflectance confocal microscopic evaluation of nonmelanocytic lip lesions. Lasers Med. Sci..

[55] Georgescu S.R., Mitran C.I., Mitran M.I., Caruntu C., Caruntu A., Lupu M., Matei C., Constantin C., Neagu M. (2020). Tumour microenvironment in skin carcinogenesis. Tumor Microenvironments in Organs.

[56] Hartmann D., Krammer S., Bachmann M.R., Mathemeier L., Ruzicka T., Bagci I.S., von Braunmühl T. (2018). Ex vivo confocal microscopy features of cutaneous squamous cell carcinoma. J. Biophotonics.

[57] Ahlgrimm-Siess V., Cao T., Oliviero M., Hofmann-Wellenhof R., Rabinovitz H.S., Scope A. (2011). The vasculature of nonmelanocytic skin tumors on reflectance confocal microscopy. Arch. Dermatol..

[58] Skobe M., Rockwell P., Goldstein N., Vosseler S., Fusenig N.E. (1997). Halting angiogenesis suppresses carcinoma cell invasion. Nat. Med..

